# BCL2 inhibition in acute and chronic pancreatitis — is there a therapeutic perspective?

**DOI:** 10.1038/s41420-026-03179-8

**Published:** 2026-05-30

**Authors:** Jacek J. Litewka, Monika A. Jakubowska, Wei Huang, Pawel E. Ferdek

**Affiliations:** 1 Department of Cell Biology, Faculty of Biochemistry, Biophysics and Biotechnology, Jagiellonian University, Kraków, Poland; 2 Doctoral School of Exact and Biological Sciences, Jagiellonian University, Kraków, Poland; 3 Malopolska Centre of Biotechnology, Jagiellonian University, Kraków, Poland; 4 West China Centre of Excellence for Pancreatitis, Institute of Integrated Traditional Chinese Medicine and Western Medicine, West China-Liverpool Biomedical Research Centre, West China Hospital, Sichuan University, Chengdu, China

**Keywords:** Acute pancreatitis, Chronic pancreatitis, Apoptosis

## Abstract

Necrotic death of pancreatic acinar cells leads to premature activation of digestive enzymes and is a central driver of inflammation in acute pancreatitis (AP). In contrast, apoptosis is a non-lytic, regulated form of cell death that prevents the release of intracellular content and is therefore considered less damaging. The induction of the intrinsic apoptotic pathway is tightly regulated by the balance between pro- and anti-apoptotic members of the BCL2 protein family. Pharmacological inhibitors of anti-apoptotic BCL2 proteins – BH3 mimetics – have thus emerged as tools to therapeutically shift cell fate from necrosis to apoptosis. Early-generation BH3 mimetics such as HA14-1 and BH3I-2′ used to validate this concept were hindered by limited potency and significant off-target effects, including disruption of calcium homeostasis. More selective and potent second-generation agents – particularly Navitoclax (ABT-263) and Venetoclax (ABT-199) – have overcome these limitations, with Venetoclax now approved for clinical use in haematological malignancies. Here, we explore the therapeutic potential of BCL2 inhibitors in pancreatic diseases, with a focus on AP and a comparison to chronic pancreatitis (CP). In AP models, BCL2 inhibition – especially with Venetoclax – redirects cell death from necrosis to apoptosis, preserving intracellular ATP, attenuating aberrant Ca^2+^ signalling, and reducing tissue damage and inflammation, without overt toxicity to healthy acinar cells in the absence of injurious stimuli. In contrast, in CP, a disease marked by fibrosis and prevailing apoptotic loss of parenchyma, BCL2 inhibition provides no benefit and may exacerbate acinar atrophy and fibrogenesis. We discuss the mechanistic basis of these divergent effects, including the role of BCL2 proteins in cell death regulation and calcium dynamics. Finally, we highlight future directions, including the rationale for clinical testing of Venetoclax in AP, and discuss cautionary considerations for its use in CP, particularly in patients with coexisting conditions such as leukaemia, who may already be receiving BH3 mimetics.

## Facts

- Selective inhibition of BCL2 shifts acinar cell fate from necrosis towards apoptosis in acute pancreatitis, preserving ATP, reducing pathological Ca^2+^ signalling and limiting tissue injury - challenging the long-standing view that, in this disease, cell death should merely be prevented rather than therapeutically redirected.
- The striking divergence between acute and chronic pancreatitis reveals that pro-apoptotic therapies are beneficial only within a narrow pathophysiological window, dependent on disease stage and mitochondrial context.
- The immunomodulatory effects of BCL2 inhibition, including lymphocyte depletion, add an important layer of complexity in pancreatitis, where early hyperinflammation frequently overlaps with evolving immunosuppression and infection risk.
- Could repurposing of Venetoclax enable short-term, carefully timed intervention that provides clinical benefit in selected cases of acute pancreatitis while maintaining an acceptable safety profile?

## Introduction: BCL2 family and BH3 mimetics

**Fig. 1:**
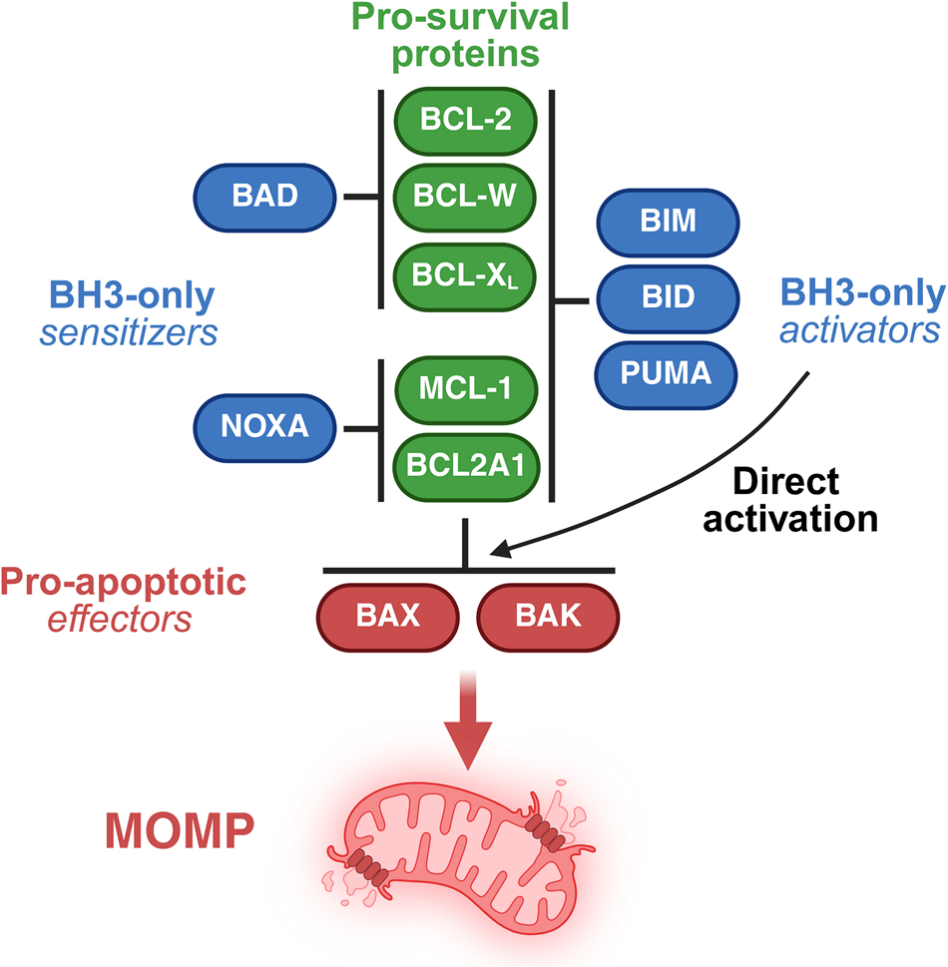
Schematic representation of the regulatory interactions within the BCL2 protein family. The BCL2 family of proteins governs the intrinsic apoptotic pathway through a tightly regulated balance between pro-survival and pro-apoptotic members. Anti-apoptotic (green) proteins (BCL-2, BCL-w, BCL-xL, MCL-1, and BCL2A1) inhibit the pore-forming pro-apoptotic effectors BAX and BAK (red), thereby preventing mitochondrial outer membrane permeabilisation (MOMP). BH3-only (blue) proteins function as upstream regulators and can be divided into two subgroups: sensitizers (e.g., BAD, NOXA), which neutralise anti-apoptotic BCL2 proteins, and activators (e.g., BIM, BID, PUMA), which can both antagonise pro-survival members and directly activate BAX and BAK.

The BCL2 protein family plays a central role in regulating the intrinsic (mitochondrial) apoptotic pathway by controlling mitochondrial outer membrane permeabilisation (MOMP) – often considered a point of no return in the execution of cell death [1, 2]. This family comprises three functional subgroups: (i) pro-survival proteins (e.g. BCL2, BCL-xL, MCL-1), (ii) pro-apoptotic effector proteins (BAX and BAK), and (iii) BH3-only proteins, which act as sensors of cellular stress that can be functionally categorised as either direct activators (directly activate BAX and BAK), or sensitizers (bind to and neutralise anti-apoptotic BCL2 family proteins without directly activating BAX/BAK) (Fig. 1) [3, 4]. Under homoeostatic conditions, BAX and BAK are present in inactive monomeric forms in the cytosol or loosely associated with intracellular membranes, including mitochondrial and endoplasmic reticulum (ER) membranes, likely in dynamic equilibrium between the cytosolic and membrane-associated pools [5–7]. The activation of these pro-apoptotic proteins requires a conformational change, typically induced by direct interaction with specific activator BH3-only proteins (such as BID, BIM, or PUMA), which triggers their oligomerisation and insertion of BAX/BAK complexes into the mitochondrial outer membrane [8–10]. Conversely, sensitizer BH3-only proteins function solely by binding to anti-apoptotic BCL2 proteins, thereby counteracting the latter’s ability to bind BAX and BAK. Once activated, BAX and BAK form pores in the mitochondrial membrane, allowing the release of cytochrome c and other pro-apoptotic factors, thereby initiating caspase activation and apoptosis [1, 11], although the detailed mechanism of cytochrome c release is still a subject of debate [12, 13]. Importantly, anti-apoptotic BCL2 family members neutralise this process at two levels: they can (1) sequester BH3-only proteins to prevent them from activating BAX/BAK, and (2) bind and inhibit already activated BAX/BAK to block their oligomerisation and MOMP [8, 9, 14]. This layered regulation provides a robust survival mechanism in healthy cells.

A key concept arising from this dynamic is ‘mitochondrial priming’— that is the extent to which a cell’s pro-survival BCL2 proteins are already engaged in sequestering BH3-only proteins [15]. Cells that are ‘primed for death’ have little remaining buffering capacity and are therefore highly sensitive to further apoptotic stimuli or BCL2 inhibitors / BH3 mimetics, which can displace BH3-only proteins or free activated BAX/BAK, pushing the cell over the apoptotic threshold [15, 16]. This concept has critical implications for both cancer therapy and certain inflammatory conditions, such as acute pancreatitis (AP), where tipping the balance towards controlled apoptosis may be therapeutically advantageous.

**Fig. 2:**
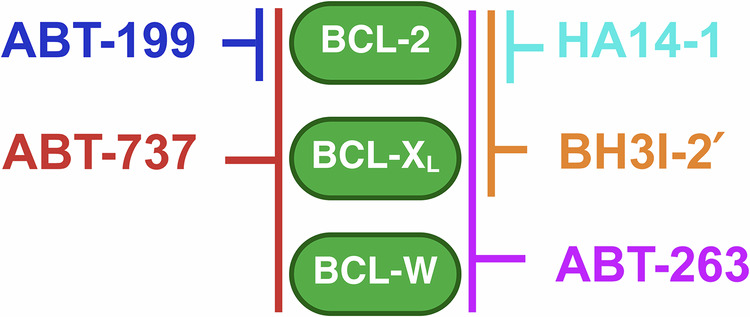
The activity of BCL-2 inhibitors against individual members of the BCL-2 protein family. The schematic illustrates the selectivity profiles of five BCL-2 family protein inhibitors: ABT-199 and HA14-1 selectively target BCL-2; BH3I-2′ targets both BCL-2 and BCL-XL; and ABT-737 and ABT-263 broadly inhibit all three proteins.

BCL2 inhibitors or BH3 mimetics are small-molecule compounds that mimic the action of BH3-only proteins by binding to the hydrophobic groove of pro-survival BCL2 family members, thus displacing pro-apoptotic partners and triggering cell death [17, 18]. Early-generation BH3 mimetics provided proof-of-concept but came with significant limitations. HA14-1, first reported in 2000, was a non-peptidic BCL2 antagonist that could disrupt BCL2–BAX interactions (Fig. 2) and induce apoptosis [19, 20]. However, HA14-1’s lack of selectivity led to off-target effects—notably causing Ca^2+^ store depletion and ER stress [21, 22]. Similarly, the compound BH3I-2′ (and its predecessor BH3I-1), identified through chemical library screens, was a low-micromolar affinity inhibitor of BCL-xL/BCL2 (Fig. 2) [23, 24]. BH3I-2′ and HA14-1 both provoked global cytosolic Ca^2+^ elevations in pancreatic acinar cells, an effect now understood to depend on BAX: as these drugs displace BAX from its complexes with BCL2, BAX may be able to insert into the ER membrane or otherwise trigger Ca^2+^ leakage [17, 22, 25]. This led to abnormal, sustained cytosolic Ca^2+^ elevations that contributed to cell injury and death [22, 25]. The calcium-destabilising properties of early BH3 mimetics confounded their usefulness and raised concerns about their safety in exocrine pancreas contexts, where cytosolic Ca^2+^ overload is itself a notorious driver of pathology [17].

The development of second-generation BH3 mimetics overcame many of these hurdles. A landmark advance was ABT-737, a rationally designed BH3 mimetic that bound with high affinity to BCL2, BCL-xL, and BCL-w [26, 27]. ABT-737 induced apoptosis potently in various models of cancer, validating the drugability of BCL2 family proteins [26]. However, ABT-737 was not orally bioavailable, and its broad target profile foreshadowed on-target side effects, such as thrombocytopenia, due to BCL-xL inhibition in platelets [28, 29]. Navitoclax (ABT-263) was developed as an orally-administrable analogue of ABT-737, retaining activity against pro-survival BCL2/BCL-xL/BCL-w (Fig. 2) [30, 31]. While Navitoclax showed clinical activity in lymphoid malignancies, its dose-limiting toxicity to platelets proved a significant challenge, attributable to the crucial role of BCL-xL in platelet survival [32–35]. The breakthrough came with Venetoclax (ABT-199), a BH3 mimetic engineered to be highly selective for BCL2 over BCL-xL (Fig. 2) [36, 37]. Venetoclax exhibits sub-nanomolar binding to BCL2 and sparing of BCL-xL, achieving robust apoptosis induction in BCL2–dependent tumour cells while largely avoiding platelet toxicity [36, 37]. Approved by the US Food & Drug Administration in 2016 as the first BH3 mimetic in clinical practice (initially for relapsed chronic lymphocytic leukaemia) [38], Venetoclax has since revolutionised treatment of certain leukaemias and lymphomas [39, 40]. In contrast to the early-generation, these newer BCL-2–inhibiting agents show no gross disruption of intracellular Ca^2+^ signalling in healthy pancreatic acinar cells [41, 42]. Table 1 provides a comparative summary of key BH3 mimetics, from early prototypes to current drugs, highlighting their targets, apoptotic potency, off-target effects, and documented impact in pancreatic models.

**Table 1:** Comparison of selected BH3 mimetics (BCL2 inhibitors) and their properties.

Year	BH3 mimetic	Primary targets	Apoptotic potency	Off-target effects Notable side effects	Effects in pancreatic models
Early 2000s	HA14-1	BCL2	EC_50_ of ~23 µM (PBMCs from CLL patients) [112]	● Inhibition of the SERCA pump and the depletion of the ER Ca^2+^ stores [21] ● Ca^2+^-dependent cytotoxicity [22, 25]	● Acinar cell death with Ca^2+^ dysregulation [22, 25] ● Not specific enough for therapeutic use [21, 113]
	BH3I-2′	BCL-xL BCL2	Induces apoptosis at ~25–30 µM (cancer cell lines) [114]	● BAX-dependent ER Ca^2+^ release [25] ● Disrupted mitochondrial functions (high doses) [21]	● Acinar cell death, global Ca^2+^ elevation, necrosis [22, 25]
2005	ABT-737	BCL2 BCL-xL BCL-w	EC_50_ of 7.0 nM (CLL cells; [115]) Induces apoptosis at 100 nM (HL-60 cells; [116]) IC_50_ of 0.73–15.6 μM (thyroid carcinoma cell lines; [117])	● BCL-xL-mediated thrombocytopenia [28, 30]	● Not orally available ● Transient Ca^2+^ signals in acinar cells without a marked effect on viability [42]
2010	Navitoclax (ABT-263)	BCL2 BCL-xL BCL-w	IC_50_ of 0.06–14.75 μM (cancer cell lines; [118]) EC_50_ of <1 μM (mesothelioma cells; [119])	● Mild off-targets (e.g. some protein binding) [120] ● Severe thrombocytopenia [121]	● AP models: shifts necrosis to apoptosis, but also kills healthy cells [49] ● CP models: exacerbated fibrosis and acinar loss [49]
2013	Venetoclax (ABT-199)	BCL2	IC_50_ of <10 nM to >1 µM (AML cell lines; [122])	● Potent apoptosis and tumour lysis syndrome (due to potent apoptosis in tumours) [36, 37] ● Neutropenia [123] ● Minimal thrombocytopenia [36]	● AP models: protective (enhanced acinar apoptosis and reduced necrosis) [49] ● CP: no fibrosis worsening [49]

*AML* acute myeloid leukaemia, *AP* acute pancreatitis, *CLL* chronic lymphocytic leukaemia, *CP* chronic pancreatitis, *EC50* Half maximal effective concentration, *ER* endoplasmic reticulum, *IC50* half maximal inhibitory concentration, *PBMCs* peripheral blood mononuclear cells, *SERCA* sarcoplasmic/endoplasmic Reticulum Ca^2+^-ATPase.

## BCL2 inhibition in acute pancreatitis: shifting necrosis to apoptosis

AP is commonly initiated by etiological factors (alcohol metabolites, gallstone obstruction, etc.) that provoke excessive Ca^2+^ release and premature digestive enzyme activation in pancreatic acinar cells [43, 44].

While apoptosis is executed when BAX/BAK are liberated to permeabilise the mitochondrial outer membrane, necrotic cell death—often precipitated by extreme cellular stress or energy failure—lacks such orderly packaging, leading to cell lysis and potent inflammatory signalling [9, 45]. In the context of AP, these distinctions are critical: extensive acinar cell necrosis correlates with severe, life-threatening pancreatitis, whereas a higher proportion of apoptosis is associated with milder disease [46, 47]. Encouraging acinar cells to die by apoptosis rather than necrosis has therefore been proposed as a therapeutic strategy in AP [48]. One way to achieve this is by targeting the BCL2 family checkpoint that controls apoptotic commitment.

Recent findings have illuminated the potential of BH3 mimetics to beneficially modulate AP outcomes, demonstrating in both ex vivo and in vivo models that BCL2 inhibitors can redirect acinar cell death from necrosis to apoptosis, with protective effects [49]. In isolated mouse acinar cells stressed with high-dose cerulein (cholecystokinin analogue) or ethanol plus fatty acid (palmitoleic acid)— two clinically relevant injury stimuli—the addition of BCL2 inhibitors significantly reduced the fraction of cells undergoing necrosis while concomitantly increasing caspase-mediated apoptotic death [49]. In this study, both Navitoclax and Venetoclax induced this shift in cell death mode, resulting in improved overall cell viability. Notably, cells treated with toxic stimuli in the presence of these inhibitors maintained higher ATP levels compared to toxin alone. ATP preservation is a hallmark of apoptosis (which requires energy) and contrasts with necrosis, where ATP is rapidly depleted [43, 49]. In AP, BCL2 inhibition may interrupt the vicious cycle of injury by preventing bioenergetic collapse, sustaining ATP, and favouring apoptosis over necrosis. Importantly, BCL2 inhibitor treatment also attenuated the upstream pathological signalling that leads to necrosis. One key observation was the blunting of aberrant intracellular Ca^2+^ dynamics in challenged acinar cells when co-treated with Venetoclax or Navitoclax [49]. Excessive and sustained rises in cytosolic Ca^2+^ are known triggers of necrosis in AP, causing mitochondrial permeability transition and ATP depletion [50]. In the experiments, control acinar cells exposed to cerulein or ethanol with fatty acid exhibited characteristic Ca^2+^ overload and oscillatory dysregulation [51]; strikingly, those receiving a BH3 mimetic showed more moderated Ca^2+^ responses [49]. This effect may stem from direct disruption of BCL2’s modulation of ER Ca^2+^ channels.

Under physiological conditions, anti-apoptotic BCL2 family members also regulate intracellular Ca^2+^ signalling. At the ER membrane, BCL2 and BCL-xL interact with inositol 1,4,5-trisphosphate receptors (IP_3_Rs) and ryanodine receptors (RyRs), thereby restraining spontaneous or excessive Ca^2+^ release into the cytosol [52–55]. In pancreatic acinar cells, this likely helps maintain Ca^2+^ homeostasis and limit sustained cytosolic Ca^2+^ elevations that promote mitochondrial dysfunction [43, 45, 50].

During acute pancreatitis, however, supramaximal stimulation and toxic metabolites overwhelm this regulatory system, causing sustained ER Ca^2+^ release, mitochondrial Ca^2+^ overload, permeability transition pore opening, and ATP depletion [43–45, 50, 51, 56, 57]. Early-generation BH3 mimetics such as HA14-1 and BH3I-2′ further destabilised this balance by depleting ER Ca^2+^ stores and inducing pathological cytosolic Ca^2+^ elevations in pancreatic acinar cells [17, 21, 22, 25]. In contrast, selective BCL2 inhibition with Venetoclax does not markedly perturb basal Ca^2+^ signalling in healthy acinar cells [41, 42]. Moreover, BCL2 loss or inhibition has been linked to enhanced plasma membrane Ca^2+^-ATPase (PMCA) activity, accelerating Ca^2+^ extrusion from the cytosol [42, 58]. This faster clearance may reduce the amplitude and duration of pathological cytosolic Ca^2+^ elevations, thereby altering downstream calcium-dependent injury pathways. Thus, in AP, selective BCL2 inhibition may attenuate sustained Ca^2+^ overload not by simply blocking ER Ca^2+^ release, but by shifting the balance away from the prolonged pathological Ca^2+^ plateau and towards more efficient Ca^2+^ clearance [42, 49, 56, 58].

Venetoclax treatment was also associated with increased pancreatic levels of S100A8/A9, a Ca^2+^-binding protein complex [49]. However, it remains unclear whether this reflects a direct consequence of BCL2 inhibition in acinar cells or a secondary effect related to altered tissue injury or inflammatory responses. We therefore consider S100A8/A9 primarily as an associated finding rather than evidence of a defined mechanistic link between BCL2 inhibition and induction of Ca^2+^-buffering or homoeostatic programmes. Nevertheless, given the known stress- and inflammation-associated roles of S100A8/A9, this observation may warrant further investigation [59].

**Fig. 3:**
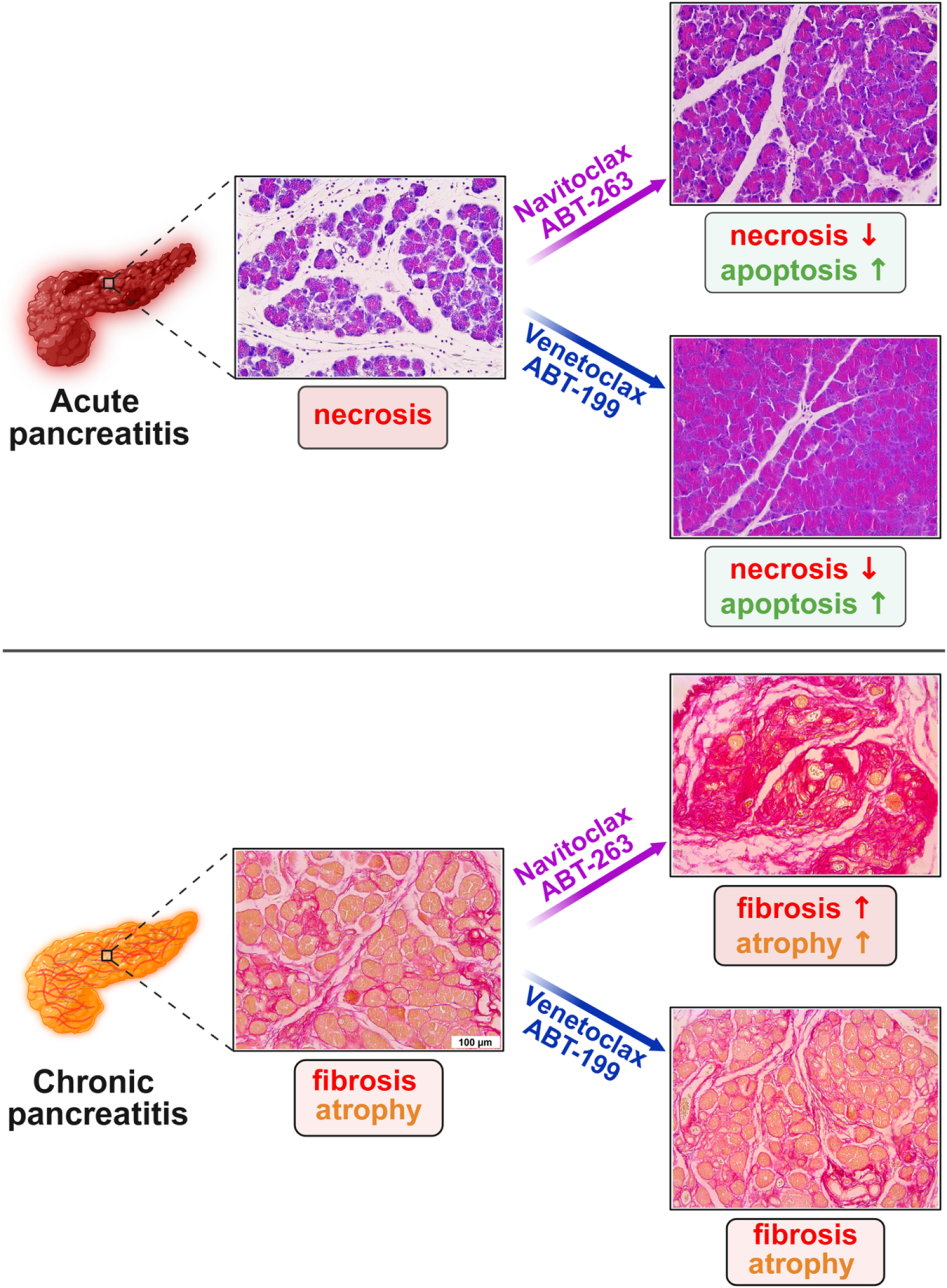
The effects of BCL2 inhibitors in models of acute and chronic pancreatitis in C57BL/6 J mice. Upper panel: Representative haematoxylin and eosin (H&E)–stained pancreatic sections from the cerulein-induced acute pancreatitis model. Pronounced oedema, inflammatory cell infiltration, and acinar cell necrosis are evident. Administration of BCL2 inhibitors markedly reduces pancreatic injury, shifting the predominant mode of cell death from necrosis to apoptosis. Lower panel: Representative Sirius Red–stained sections (fibrosis in red) from the cerulein-induced chronic pancreatitis model. Extensive tissue fibrosis and acinar atrophy are observed. No histological improvement is seen following Venetoclax treatment. In contrast, Navitoclax administration enhances fibrosis and acinar cell atrophy. The images are included here for illustrative purposes as part of a schematic summary of the effects observed in vivo; quantitative histological analyses were reported in the original experimental study [49].

The beneficial impact of BCL2 inhibitors in AP extends to whole-animal models as well. In murine AP induced by cerulein hyperstimulation or by alcohol and fatty acid [60], Venetoclax administration markedly reduced pancreatic injury. Treated mice showed less acinar necrosis and more apoptosis than untreated AP controls, confirmed by cleaved caspase-3 staining and quantitative image analysis demonstrating a higher apoptotic index and reduced necrotic area [49]. Functionally, this translated to milder pancreatitis: less oedema, inflammatory infiltration, and systemic complications. By shifting cell death towards the non-lytic pathway, BCL2 inhibition transformed what would be a severe necrotising pancreatitis into a significantly blunted inflammatory response (Fig. 3). It is noteworthy that Navitoclax produced a similar apoptotic shift and disease attenuation in these models, but with caveats discussed below. As both drugs were given before and during injury induction, their anti-necrotic effect was likely maximised. This may model early intervention in predicted severe AP, where a rapid pro-apoptotic treatment could prevent progression to fulminant necrosis.

One might recall that earlier studies had warned that disabling BCL2 and BCL-xL would worsen pancreatitis by unleashing mitochondrial dysfunction and necrosis. Broad BCL2/BCL-xL inhibition in acinar cells was reported to trigger mitochondrial depolarisation, ER Ca^2+^ store depletion, ATP loss and necrosis (more so when combined with a pancreatitis stimulus), leading to the conclusion that BCL-xL protects against necrosis by stabilising mitochondria [22, 61]. How can these findings be reconciled with the newer evidence that BCL2 inhibitor treatment is beneficial? The discrepancy likely lies in the context and specificity of the intervention. Non-selective inhibition of both BCL2 and BCL-xL, whether pharmacological or genetic, can indeed tip severely stressed cells over the edge into bioenergetic catastrophe, as BCL-xL is a potent guardian of mitochondrial physiology also in acinar cells [61]. Accordingly, broad BCL2 family inhibition, for example, with Navitoclax, may even induce apoptosis in healthy pancreatic tissue [49]. In contrast, selective targeting of BCL2 alone—as with Venetoclax—appears sufficient to promote apoptosis of vulnerable cells largely preserving tissue integrity, likely because BCL-xL remains available to support mitochondrial energetics in the surviving cells [49]. Moreover, in vivo, apoptotic cells (e.g. triggered by Venetoclax) can be efficiently cleared by phagocytes, thereby preventing secondary necrosis and fostering resolution [62]. Thus, a nuanced picture emerges: a calibrated inhibition of anti-apoptotic defences may induce ‘useful apoptosis’ in AP, whereas broader or excessive inhibition, or intervention when clearance capacity is limited, may instead exacerbate necrosis. The data support that Venetoclax—owing to its selectivity and ability to induce controlled apoptosis—may represent a novel therapeutic avenue in acute pancreatitis by fundamentally altering cell death in favour of a less injurious outcome [49].

One further consideration is that even selective BCL2 inhibition may not be immunologically neutral. This may be particularly relevant in severe AP, where early hyperinflammation can coexist with evolving immune dysfunction, as discussed in greater detail later.

## Contrasting chronic pancreatitis: apoptosis, fibrosis and the limits of BCL2 inhibition

Chronic pancreatitis (CP) is characterised by ongoing inflammation, acinar cell attrition, fibrosis, and tissue remodelling over months to years [63, 64]. The pathological hallmark of CP is fibrotic replacement of exocrine tissue, driven by activated pancreatic stellate cells (PSCs) in response to injury [65, 66]. Given the success of BCL2 inhibition in promoting apoptosis in AP, one might wonder if a similar approach could ameliorate CP by removing damaged cells or even by targeting PSCs, as previous in vitro studies have suggested BCL2-linked survival pathways help sustain the activated PSC phenotype, with BCL2 downregulation being associated with reduced PSC proliferation and migration as well as enhanced PSC apoptosis [67, 68]. However, evidence to date indicates that BCL2 inhibitors are at best ineffective in CP and at worst counterproductive.

In experimental mouse CP models, co-administration of Navitoclax or Venetoclax during the chronic injury phase yielded strikingly different outcomes compared to AP. Venetoclax was largely neutral, neither significantly improving nor worsening chronic pancreatic injury. In particular, fibrosis (assessed by collagen deposition) was unchanged compared to untreated CP [49]. By contrast, Navitoclax substantially aggravated chronic pancreatic damage (Fig. 3). Mice with CP that received Navitoclax showed greater acinar cell loss, more severe inflammatory and fibrotic changes, and higher overall pathology scores than CP controls [49]. Collagen staining revealed that Navitoclax drove significantly more fibrosis, even in the pancreatic tissue not subjected to the CP induction. Proteomic profiling supported these findings, as Navitoclax-treated CP samples showed upregulation of extracellular matrix proteins and pro-fibrotic pathways, along with downregulation of protein synthesis machinery, consistent with irreversible acinar loss [49]. Venetoclax-treated CP samples exhibited only mild changes, suggesting a more tolerable profile that does not exacerbate fibrosis.

Mechanistically, promoting apoptosis in CP faces two major limitations. First, because apoptosis already predominates in chronic pancreatic injury, pushing cells harder into apoptosis yields diminishing returns. Human CP tissue shows high rates of acinar apoptosis and a relative paucity of necrosis [69, 70]. In the adult pancreas, regeneration relies mainly on cellular plasticity and phenotypic interconversion, particularly of acinar cells, which can adopt progenitor-like states, rather than by a dedicated pool of classical stem cells [71, 72]. Yet the fibrogenic environment continuously eliminates acinar cells, and the few that attempt regeneration often succumb to mitochondrial dysfunction and stress-induced apoptosis. Thus, a pro-apoptotic therapy is unlikely to improve function; instead, it risks overshooting and removing too many cells that might otherwise sustain some exocrine capacity.

Second, the fibrotic response in CP can override any direct anti-cellular effects on PSCs. Notably, in vitro tests revealed that Navitoclax can kill activated PSCs, which in theory should reduce fibrogenesis [49]. Yet in vivo, ongoing acinar cell loss and persistent injury signals can rapidly recruit and activate new PSCs (or stimulate the proliferation of existing ones) [73, 74], negating any transient reduction in stellate cell numbers. In fact, by causing additional acinar cell apoptosis and tissue damage, Navitoclax likely fuels a feed-forward pro-fibrotic loop. Injured and dying acinar cells release PSC-activating mediators, including TGF-β, PDGF, and TNF-α [75–77], while apoptotic bodies and other injury-derived products may further promote PSC activation through inflammatory and phagocytic signalling pathways [78, 79]. Thus, even if BCL2 inhibition transiently reduces PSC viability, this effect may be outweighed in vivo by the continued generation of pro-fibrotic cues from the injured parenchyma. BCL2 inhibition may also affect PSC behaviour beyond cell survival alone, for example, by altering stress responses or activation state, although this remains insufficiently explored. As a result, eliminating PSCs, without addressing ongoing pro-fibrotic stimuli (ongoing injury, cytokines), merely creates space that is soon filled by newly emerging matrix-producing cells. In the pancreas, chronic injury tends to persist (patients often have risk factors like alcohol or genetic predispositions that continue), and thus, pro-apoptotic therapy fails to revert fibrosis. The liver has taught us a parallel lesson: inducing apoptosis in hepatic stellate cells can reduce fibrosis in some circumstances, but only if the injurious trigger is removed and the timing is right [80, 81].

Why BCL2 inhibitors have such divergent effects in AP versus CP also depends on the delicate balance of mitochondrial regulation and Ca^2+^ dynamics controlled by BCL2 family proteins, and how this balance changes in the pathophysiological context. During AP, pathological Ca^2+^ signals (triggered by bile acids, fatty acid ethyl esters, etc.) provoke mitochondrial Ca^2+^ overload, opening of the permeability transition pore, and collapse of ΔΨ_m_ [50, 51, 82]. This leads to ATP depletion and necrosis unless checked. In this acute scenario, introducing a BCL2 inhibitor such as Venetoclax effectively lowers the threshold for initiating mitochondrial apoptosis (via BAX/BAK activation) before catastrophic mitochondrial failure. Apoptosis can be seen as a pre-emptive mitochondrial-controlled demolition: by allowing cytochrome c release and caspase activation, the cell is taken offline in a tidy manner, preventing the full-on bioenergetic crash that results in necrosis [9, 83]. Moreover, once caspases are active, they cleave a host of substrates, potentially including Ca^2+^ signalling proteins, thereby dampening further Ca^2+^ release and cutting short the feed-forward loops that sustain necrosis [84, 85]. The net effect is that the cell dies sooner, but more quietly, and its remains are rapidly phagocytosed—a net win in the context of an overly exuberant inflammatory injury.

In CP, however, the mitochondrial and Ca^2+^ dynamics differ. Over the chronic course, many acinar cells have adapted or succumbed; those that remain may express higher levels of BCL-xL as a protective adaptation (as observed in some models) [61]. Mitochondrial dysfunction in CP is more linked to chronic oxidative stress and metabolic impairment than acute Ca^2+^ overload, with oxidative stress likely reflecting persistent injury, inflammation, impaired antioxidant defences, and continued exposure to factors such as alcohol and smoking [46, 86, 87]. Theoretically, adding a BCL2 inhibitor to cells under oxidative stress may either unmask latent apoptotic priming and trigger apoptosis, or have limited benefit if survival has shifted to other anti-apoptotic proteins, with the net effect depending on the dominant BCL2-family dependency and the bioenergetic state of the cell [16, 88]. Whether this results in a therapeutically useful effect, therefore, remains context-dependent. However, when established CP is considered, tipping some cells into apoptosis with a BCL2 inhibitor is unlikely to salvage the situation, because mitochondria are already damaged in many cells, and caspase pathways might be chronically active at low levels. Instead, what the chronically inflamed pancreas needs is halting of the fibrotic cycle and promotion of regeneration, neither of which is achieved by inducing more cell death.

An illustrative outcome from these studies is that indiscriminate apoptosis induction is not a one-size-fits-all remedy for pancreatic diseases. In CP, the target is not to eliminate cells (as most functional acini are already gone) but rather to modulate the inflammatory-fibrotic milieu and promote regeneration. BCL2 inhibitors do not address the core drivers of fibrosis, such as TGF-β signalling, nor do they rescue remaining acinar cells from stress; instead, they may simply hasten their demise. Therefore, while BCL2 inhibition could potentially be a compelling strategy for AP, its application in CP appears unwarranted and potentially harmful.

## Immunosuppressive Impact of BCL2 inhibitors

It is also important to consider that BCL2 inhibitors can affect the immune system. This issue is directly relevant to pancreatitis, because any protective effect on pancreatic acinar cell death must be balanced against the possibility of aggravating disease-associated immune dysfunction. BCL2 plays a crucial role in lymphocyte survival; accordingly, ABT-737 has been shown to cause significant depletion of T lymphocytes (both CD4^+^ and CD8^+^) and B cells in vivo, leading to impaired cytotoxic T-cell responses and diminished memory B-cell persistence [89]. Notably, these immunosuppressive effects were potent enough that ABT-737 prolonged pancreatic islet allograft survival in mice by preventing immune rejection [89].

In severe AP, an initial hyperinflammatory response is frequently accompanied or followed by immunosuppression, partly driven by widespread early lymphocyte apoptosis [90], an effect also well documented in sepsis. If BCL2 inhibitors drive additional apoptosis in immune cells, such as CD8^+^ T cells, they may further worsen lymphopenia and weaken host defences. Evidence from sepsis models shows that overexpression of BCL2 protects against lymphocyte apoptosis and improves survival [91, 92], suggesting that BCL2 inhibition may promote immune cell loss. Clinically, patients receiving Venetoclax for leukaemia often experience cytopenias, including neutropenia, and increased infection rates have been observed in some treatment regimens [93]. This implies that BCL2 blockade can increase susceptibility to infections—a critical concern in necrotising pancreatitis, where secondary infections such as infected pancreatic necrosis or sepsis are major contributors to mortality.

**Fig. 4:**
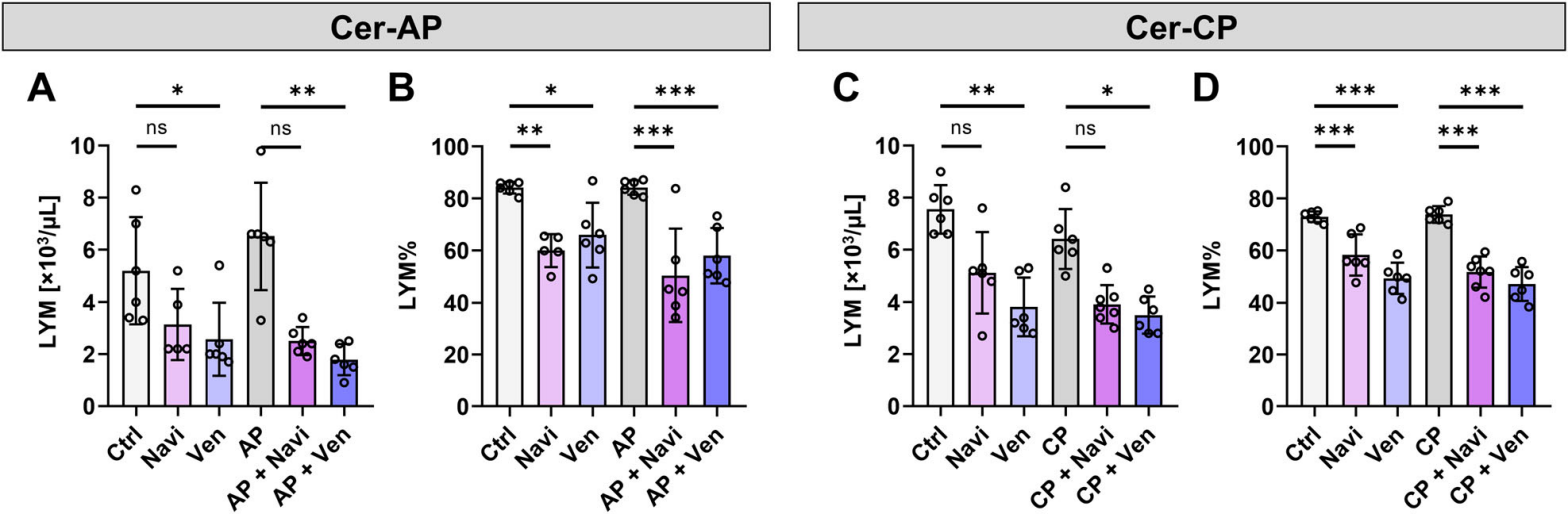
Effects of Navitoclax and Venetoclax on lymphocyte counts in acute and chronic pancreatitis. **A** Absolute lymphocyte counts in the cerulein-induced acute pancreatitis (Cer-AP) model in mice; **B** and their relative abundance expressed as percentage of total leukocytes (Lym%). **C** Absolute lymphocyte counts in the cerulein-induced chronic pancreatitis (Cer-CP) model in mice; **D** and the corresponding relative values (Lym%). Data are presented as mean ± SD, with individual data points representing values from individual mice. Normality was assessed using the Shapiro–Wilk test. Absolute lymphocyte counts were not normally distributed and were analysed using the Kruskal–Wallis test with Dunn’s multiple comparisons. Lym% values showed normal distribution and were analysed using one-way ANOVA with Sidak’s multiple comparisons. *P* < 0.05 (*), *P* < 0.01 (**), *P* < 0.001 (***); ns, not significant. In the acute pancreatitis (AP) model, pancreatitis was induced by seven hourly intraperitoneal injections of cerulein (50 µg/kg). Navitoclax (Navi) or Venetoclax (Ven) (100 mg/kg, intraperitoneally) was administered 24 h before and on the day of AP induction, prior to the first cerulein injection. Animals were euthanised 24 h after induction, and blood was collected post mortem. In the chronic pancreatitis (CP) model, pancreatitis was induced by repeated cerulein injections (50 µg/kg, seven hourly injections, twice weekly) for 8 weeks. Navitoclax or Venetoclax (100 mg/kg, intraperitoneally) was administered twice weekly from weeks 5–8, 24 h prior to cerulein injections. Animals were euthanised 72 h after the final injection, and blood was collected post mortem. The data shown were obtained from experiments previously published [49].

Although Venetoclax is more selective (sparing BCL-xL) and was found to be safer than Navitoclax in preclinical models of AP [49], this selectivity should not be interpreted as immunological neutrality, because BCL2-dependent immune cell populations may still be affected. Some studies suggest that Venetoclax can be combined with immunotherapy without compromising T-cell function [94], indicating that activated T cells, which rely more on MCL1, may resist BCL2 inhibition. However, data from our laboratory obtained in mouse models of AP and CP indicate that both Navitoclax and Venetoclax are associated with reduced peripheral blood lymphocyte counts (Fig. 4A–D), an effect that is particularly evident when lymphocytes are analysed as a proportion of total leukocytes (Fig. 4B, D). In the context of AP, this finding should be regarded as a potentially relevant safety signal, particularly in later disease phases, when infection of pancreatic necrosis and sepsis become major determinants of outcome, and when alterations in lymphocyte populations may also impair the resolution phase by disturbing immune mechanisms that help restrain inflammation and support tissue recovery.

Mechanistically, this reduction in peripheral blood lymphocyte counts may reflect on-target induction of mitochondrial apoptosis in BCL2-dependent lymphocyte subsets. Previous work indicates that naïve T cells, and at least some memory T-cell compartments, depend on BCL2 for peripheral survival, whereas effector-memory populations may be relatively less sensitive because of greater reliance on alternative pro-survival proteins [95, 96]. In keeping with this, short-term Venetoclax treatment preferentially affects naïve and central-memory T-cell subsets in vivo [97]. In mice, ABT-199 (Venetoclax) also reduces multiple lymphocyte populations ex vivo, supporting the view that the lymphocyte decrease observed in our models is at least partly drug-related [98]. Therefore, in the setting of acute inflammatory disease, the net effect of BH3 mimetics will likely be immunosuppressive—especially for naïve and memory T-cell pools, which are highly BCL2-dependent and essential for mounting new immune responses [89].

Finally, it should also be acknowledged that current experimental models only partially recapitulate the complexity of human CP. In particular, repetitive cerulein administration models acinar injury and fibrosis, but does not fully capture ductal obstruction, genetic predisposition, metabolic factors, and long-standing progressive tissue remodelling [60]. While cerulein, a cholecystokinin analogue, is used to induce pancreatic inflammation experimentally, there is no evidence that high cholecystokinin levels occur in humans or cause pancreatitis [99, 100]. These limitations should be kept in mind when interpreting the lack of benefit of BCL2 inhibition in experimental CP. Nevertheless, the absence of antifibrotic efficacy, together with the worsening observed with Navitoclax, argues for caution in extrapolating pro-apoptotic strategies to chronic pancreatic injury.

## Future directions and clinical implications

The promising results with Venetoclax in preclinical AP models provide cautious encouragement for clinical translation. A key advantage is that Venetoclax is already an approved drug with a well-characterised safety profile from its clinical use in haematologic cancers since 2016 [38]. This could expedite repurposing efforts for AP. In an acute, life-threatening condition like severe pancreatitis, a fast-track approach using an existing drug is attractive, provided there is solid evidence of benefit and manageable risks. Short-term administration of Venetoclax during an AP episode could be envisioned to limit necrosis and systemic complications—for example, as an adjunct to intensive care measures in necrotising pancreatitis. Of course, careful clinical trials will be needed to assess efficacy (e.g. reduction in necrotic volume or inflammatory markers, improvement in organ failure scores) and safety in this new context. Possible adverse effects to monitor include cytopenias (neutropenia lymphopenia or thrombocytopenia). Although Venetoclax is less likely than less selective BCL2 inhibitors to cause marked thrombocytopenia because it largely spares BCL-xL, it may still affect other BCL2-dependent blood and immune cell populations [36, 101]. This issue may be particularly important in severe AP, where early hyperinflammation can overlap with evolving immune dysfunction; in such patients, further depletion of BCL2-dependent lymphocyte populations could increase susceptibility to secondary infection. Further research is needed to delineate how these drugs influence immune cell subsets during AP—for instance, whether short-term use might be immunomodulatory without irreversible lymphocyte loss, or if certain dosing strategies could spare immune function. The anti-necrotic benefits should be carefully balanced against the danger of immune compromise, with vigilant monitoring for infections. Tumour lysis–like syndromes are not relevant in AP, but one must consider that AP patients often have co-morbidities and multi-organ stress, so any pro-apoptotic agent should be used judiciously. The timing of administration in AP might also be critical – too late in the course (after extensive necrosis has occurred), and the benefit might be negligible, or the drug might struggle to penetrate poorly perfused necrotic areas. This consideration also helps explain the design of the animal study, in which BCL2 inhibitors were administered before the induction of AP. In the short murine model, this approach was used to avoid missing the likely narrow window during which modulation of cell death could still influence disease evolution before necrosis became established, whereas in human AP the clinical course is typically longer, potentially leaving a broader window for early therapeutic intervention [49, 102].

An intriguing aspect is whether Venetoclax (or similar agents) could synergise with emerging therapies targeting Ca^2+^ overload in pancreatitis. Inhibitors of Ca^2+^ influx channels (e.g., ORAI1 inhibitors such as GSK7975A or CM128) and TRPM2 channel inhibitors have shown efficacy in reducing acinar necrosis in vitro and inflammation in models of AP [103–106]. A combination approach – for instance, using a Ca^2+^ influx inhibitor to prevent the initial Ca^2+^ surge, alongside Venetoclax to ensure any cells beyond rescue undergo apoptosis—could theoretically provide an even greater protective effect. This integrated strategy targets both the trigger (calcium toxicity) and the cell death outcome.

On the other hand, the role of BCL2 inhibitors in CP or in patients with underlying chronic pancreatic damage demands caution. Given the evidence that Navitoclax worsened fibrogenesis in mice [49], one must be wary of using BH3 mimetics in CP patients outside of a carefully monitored research setting. This is particularly relevant because some CP patients might coincidentally be candidates for BCL2 inhibitor therapy due to other illnesses. For instance, an individual with advanced CP who develops chronic lymphocytic leukaemia might be placed on Venetoclax for the malignancy. Clinicians should be alert to the possibility that such treatment, while targeting the blood cancer, could have unintended consequences on the patient’s pancreatic disease. There is no clinical report yet of Venetoclax precipitating pancreatitis or accelerating CP, but awareness is warranted.

Looking forward, patient stratification will be necessary. Not all cases of AP would merit pro-apoptotic therapy; mild pancreatitis is self-limited and likely does not require intervention [102, 107]. The target population would be moderate to severe AP, where the risk of necrosis and complications is high. Biomarkers that reflect cell death modes (e.g. circulating DNA from necrotic cells vs. caspase-cleaved substrates) could potentially guide the decision to deploy such a therapy. Additionally, dosing regimens need optimisation—perhaps a short course of Venetoclax at the onset of severe AP, rather than continuous dosing, to avoid any long-term suppression of cell survival pathways.

More broadly, the concept of therapeutically redirecting cell death from necrosis towards apoptosis may also be relevant to other acute inflammatory diseases in which tissue injury and inflammatory amplification are tightly coupled [108, 109]. This may apply, for example, to acute kidney injury and acute lung injury, where lytic cell death can intensify inflammation, whereas apoptotic cell clearance is linked to resolution [110, 111]. However, whether pharmacologically promoting apoptosis would be beneficial in such settings would remain highly context-dependent and would require careful disease-specific evaluation.

## Concluding remarks

BCL2 inhibitors, Venetoclax in particular, represent a novel therapeutic concept in acute pancreatitis by pharmacologically biasing cell death towards apoptosis and away from necrosis. This paradigm shift has demonstrated preclinical benefits, including preservation of pancreatic tissue, reduction of inflammation, and improved systemic outcomes. While translating this to clinical practice will require rigorous evaluation, the door is now open for clinical trials, especially since the drug is already in our armamentarium. For chronic pancreatitis, however, the current evidence urges restraint—the complex pathology of CP likely cannot be undone by forcing more cell death. Instead, future therapies for CP may focus on anti-fibrotic and pro-regenerative strategies rather than apoptotic modulation. In the interim, clinicians should approach any use of BCL2 inhibitors in CP patients with caution, considering individual risk-benefit and monitoring for pancreatic effects. The contrasting tales of AP and CP teach us a broader lesson: successful therapeutic targeting of the BCL2 family hinges on context, timing, and the fine line between protective apoptosis and destructive cell loss. With continued research, what we learn in the extreme setting of pancreatitis might also inform the use of BCL2 inhibitors in other inflammatory or degenerative diseases where controlling how and when cells die is key.
